# Soweto Cardiovascular Research Unit: Patients with heart disease encouraged to live and eat healthily

**Published:** 2010-06

## Introduction

The Soweto Cardiovascular Research Unit partners with Adcock Ingram, through its generics portfolio team, to host workshops on disease management and cooking demonstrations on healthy eating for heart disease patients at Chris Hani Baragwanath Hospital.

The findings of the Heart of Soweto study, which was established to monitor, describe and respond to the evolving burden of heart disease in Soweto, showed that increased urbanisation has resulted in a deterioration in individuals’ previously more healthy lifestyles and diets. Urbanisation results in certain lifestyle shifts, including a decrease in physical activity, changes in diet and eating patterns, adoption of the use of tobacco, and an increase in alcohol consumption.

Several studies have been conducted to identify the barriers to successful lifestyle and dietary changes in patients with chronic diseases. These include a lack of awareness of the benefits, poor understanding of low-sodium dietary guidelines, difficulties with the taste, selection and preparation of recommended foods, lack of understanding of food labels and the lack of spousal and family support.^1^

This, coupled with the fact that the overall burden of cardiovascular disease is predicted to rise by approximately 150% in developing countries in the next 20 years^2^ prompted the Soweto Cardiovascular Research Unit to develop workshops and cooking demonstrations to promote practical healthy lifestyle choices, and to empower patients diagnosed with heart disease.

The Soweto Cardiovascular Research Unit, supported by Adcock Ingram community upliftment programmes, run regular workshops and cooking demonstrations with the objective of providing information on medical and nutritional management of chronic diseases of lifestyle to patients and their family members.

‘I give patients healthy eating guidelines, and teach them the nutritional benefits of basic food groups, what foods to eat and which to avoid, given their medical condition. Patients enjoy hands-on preparation of cost-effective meals that are “heart healthy” and based on traditional recipes’, explains dietician, Sandra Pretorius.

Prof Karen Sliwa-Hahnle, senior cardiologist and director of the Soweto Cardiovascular Research Unit says, ‘The Heart of Soweto study was developed with the aim of addressing our poor understanding of the characteristics of and burden imposed by cardiovascular disease within South Africa. The findings were alarming and one of the biggest threats identified was the lack of nutritional knowledge. We decided to develop a series of lifestyle-management workshops and cooking demonstrations, to give our heart-disease patients the skills and information on healthy lifestyle choices and food preparation so that they are able to live right and eat right, extending their life expectancy.’

‘Adcock Ingram, under the leadership of our generics team, is very excited to be partnering with the Soweto Cardiovascular Research Unit on this project. It is our company’s mission to add value to life and in this case, we want to empower patients suffering with heart disease with skills on how to manage their chronic diseases and improve their lifestyles.

We remain committed to doing this by encouraging family members to attend the workshops and cooking demonstrations, so that they are not only able to support their affected family members but also assist them in making healthier lifestyle choices and prepare meals that are healthy and nutritious’, says Mike Mabasa, head of corporate affairs and communications at Adcock Ingram.
